# Supplementary material for: Structural determinants of Rab11 activation by the guanine nucleotide exchange factor SH3BP5
Source: Nat Commun. 2018 Sep 14;9:3772. doi: 10.1038/s41467-018-06196-z (PMC6138693; doi:10.1038/s41467-018-06196-z)
Supplement: Supplementary file 1 — Supplementary Information [file 41467_2018_6196_MOESM1_ESM.pdf]

Supplementary information for

Structural determinants of Rab11 activation by the Guanine Nucleotide Exchange Factor SH3BP5

Meredith L Jenkins[1], Jean Piero Margaria[2], Jordan TB Stariha[1], Reece M Hoffmann[1], Jacob A McPhail[1], David J Hamelin[1], Martin J Boulanger[1], Emilio Hirsch[2], John E. Burke[1*]

[1]Department of Biochemistry and Microbiology, University of Victoria, Victoria, British Columbia, V8W 2Y2, Canada

[2]Department of Molecular Biotechnology and Health Sciences. Molecular Biotechnology Center, University of Turin, Via Nizza 52, 10126 Torino, Italy

Supplementary Figures

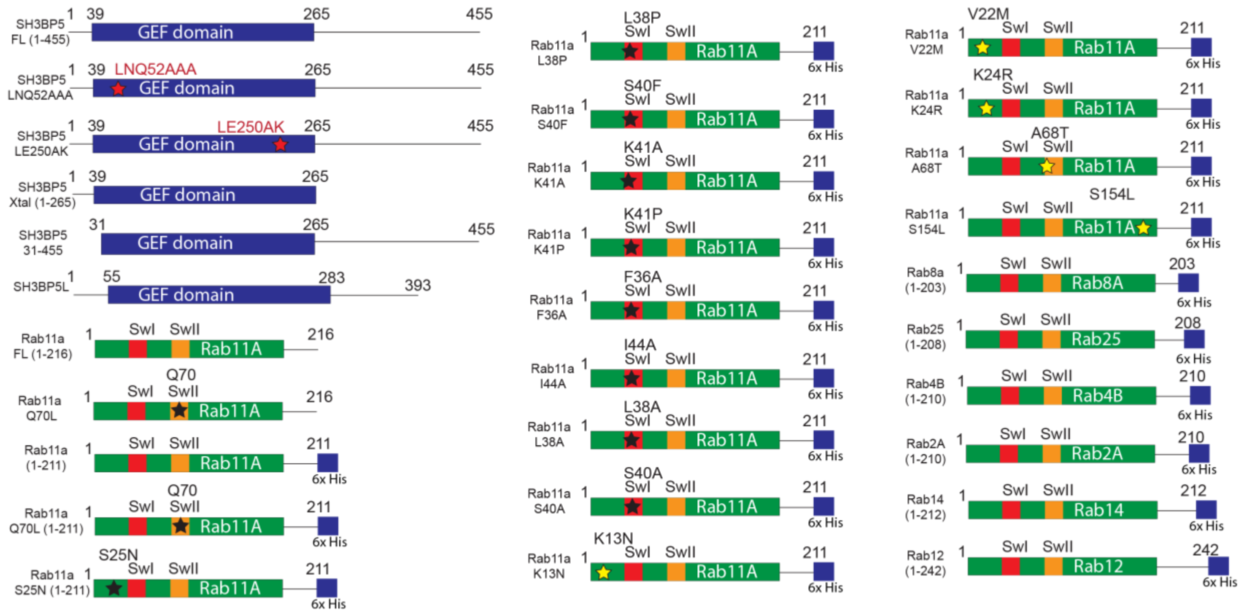

Supplementary Figure 1. List of all purified SH3BP5 and Rab11 constructs. Domain representation of all constructs used in this paper is listed above. Clinically relevant mutations are highlighted with a yellow star.

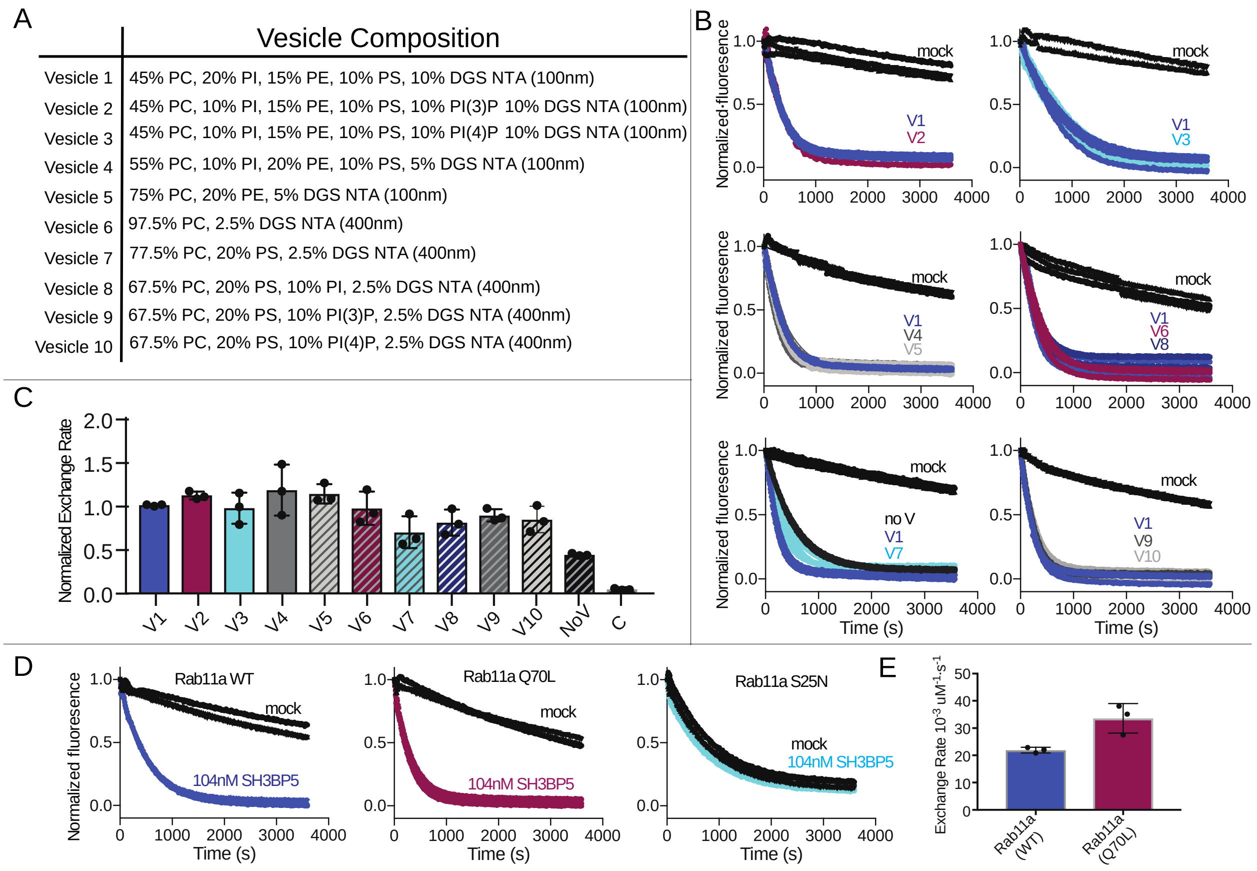

Supplementary Figure 2. GEF assays of Rab11 in the presence of membrane.

(A) Lipid compositions in the different membrane vesicles used in this experiment, different vesicles are abbreviated as V1, V2, etc.

(B) In vitro GEF activity exhibited towards Rab11A in the presence and absence of membrane. Composition of vesicles does not affect GEF activity *in vitro*. Nucleotide exchange was monitored by measuring the fluorescent signal during the SH3BP5(1-455) (52nM) catalyzed release of Mant-GDP from 4µM of Rab11A-His_6 in the presence of 100µM GTPγS. Membrane was present at a final concentration of 0.2mg/ml (V1, V2, V3, V4, V5) or 0.4mg/ml (V6, V7, V8, V9, V10). Fluorescent measurements were completed every 11 sec for a total of 60 min (Excitation λ = 366nm; Emission λ = 443nm). GEF activity was monitored in the presence of varying membrane

compositions, with total GEF activity normalised to the Rab11 GEF activity in the presence of V1-vesicles.

(C) Quantification of GEF activity in the presence and absence of different Ni-NTA membranes. All experiments were normalized to the exchange rate of V1.

(D) In vitro GEF activity exhibited towards Rab11A mutants Rab11A(Q70L) and Rab11A(S25N). A measurable rate of GEF activity on Rab11A(S25N) was not obtained as the basal state released nucleotide at the same rate as SH3BP5 bound.

(E) Quantification of GEF activity on Rab11A WT and Rab11A Q70L. For all panels, error bars show SD (n=3).

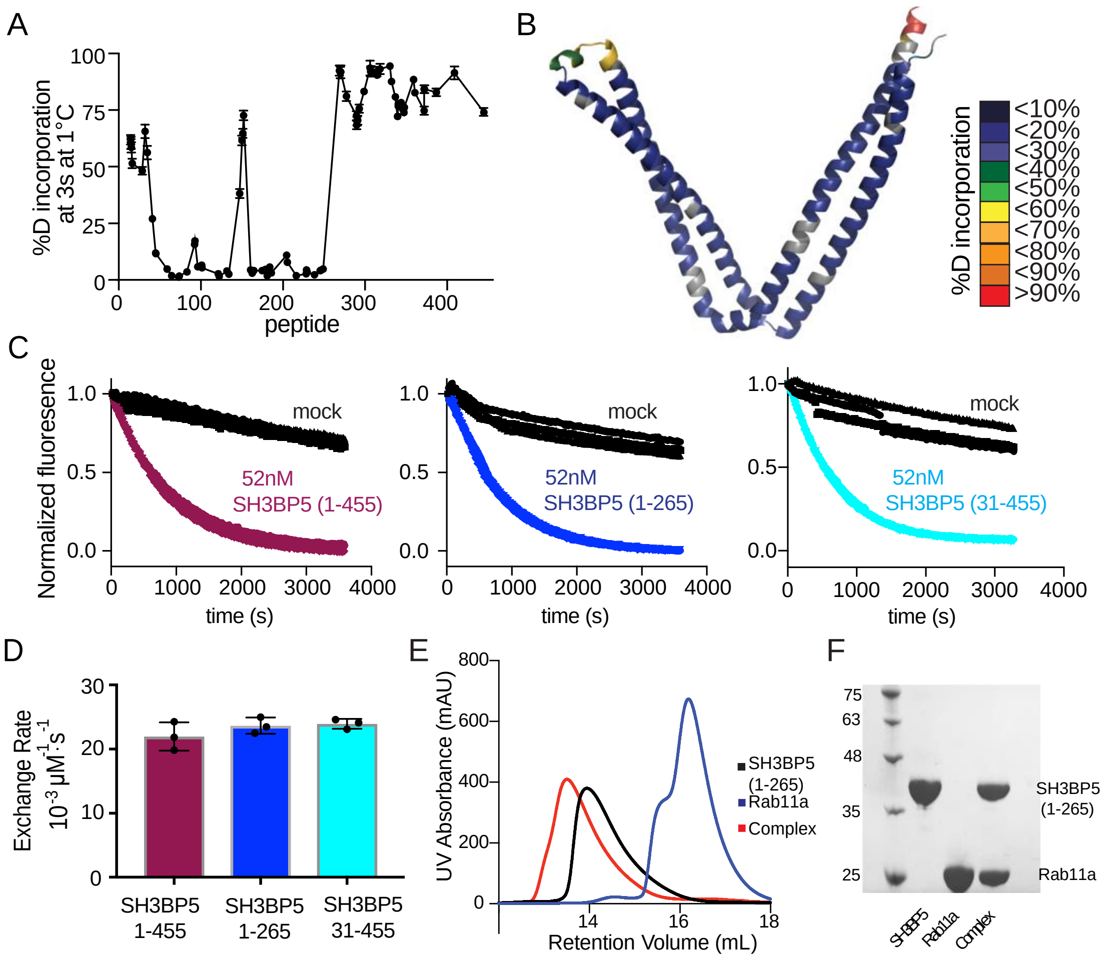

Supplementary Figure 3. HDX-MS to map the ordered regions of SH3BP5, and GEF activity of SH3BP5 (1-265 vs full length).

(A) Hydrogen deuterium exchange levels for full length SH3BP5 (1-455) after 3 seconds of deuterium exposure at 1^{0}C. Every point represents the central residue of an individual peptide vs the % deuterium incorporation.

(B) HDX data from panel A mapped on the structure of SH3BP5.

(C) GEF assays of SH3BP5 (1-455), SH3BP5 (31-455), and SH3BP5 (1-265) showed that the crystal construct and the construct used in HDX-MS experiments has the same activity as the full-length construct.

(D) Quantification of the GEF data in panel C. For all panels, error bars show SD (n=3).

(E) Size Exclusion Chromatography (SEC) trace of the SH3BP5:Rab11A complex. Apo proteins, and proteins mixed at a 1:1 molar ratio were subject to SEC on a Superdex 200 increase 10/300 column. Rab11 and SH3BP5 co-eluted in a new peak, indicating complex formation.

(F) An SDS-PAGE gel of each SEC peak is shown (15% gel run at 200V for 45 min and stained with Coomassie Brilliant Blue dye).

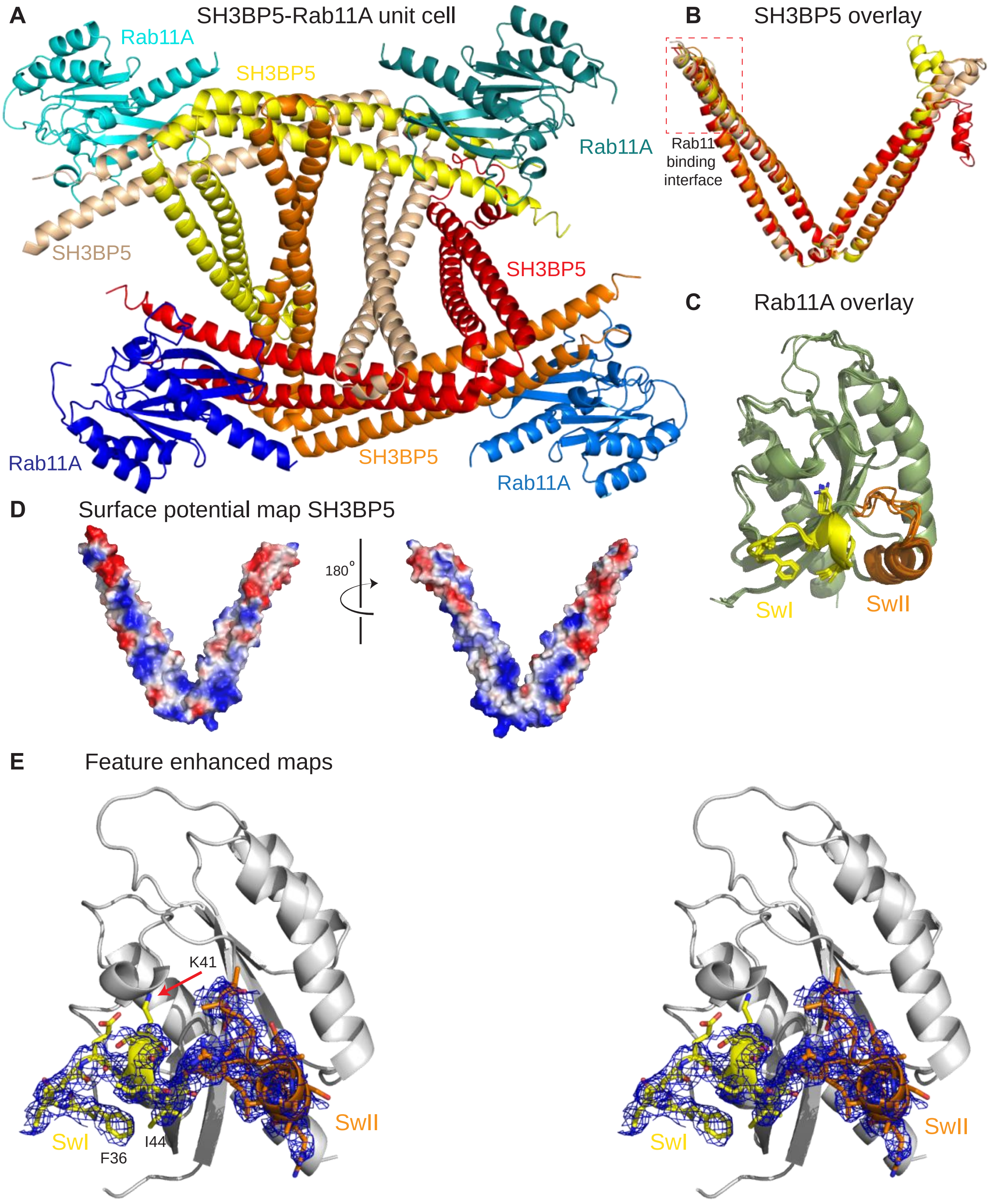

A
SH3BP5-Rab11A unit cell
Rab11A
SH3BP5
Rab11A
SH3BP5
SH3BP5
Rab11A
SH3BP5
Rab11A
B
SH3BP5 overlay
Rab11
binding
interface
C
Rab11A overlay
SwI
SwII
D
Surface potential map SH3BP5
180°
E
Feature enhanced maps
K41
SwI
F36
I44
SwII
SwI
SwII

Supplementary Figure 4. Crystallographic unit cell of SH3BP5 bound to Rab11.

(A) The asymmetric unit of SH3BP5-Rab11 is composed of four complexes each of both SH3BP5 and Rab11. The different copies are colored according to the legend.

(B) Overlay of the four SH3BP5 copies in the asymmetric unit reveal highly conserved similarity at the Rab11 binding interface, with a large degree of conformational variability at the hinge between helix α2 and helix α3.

(C) Overlay of the four Rab11 copies in the asymmetric unit reveal a highly conserved Rab conformation throughout the asymmetric unit.

(D) Surface potential map of SH3BP5 generated using APBS[1].

(E) Stereo image of the feature enhanced map of switch I and switch II generated in Phenix[2] contoured at 1.0σ (blue mesh). Switch I and II residues are shown as sticks.

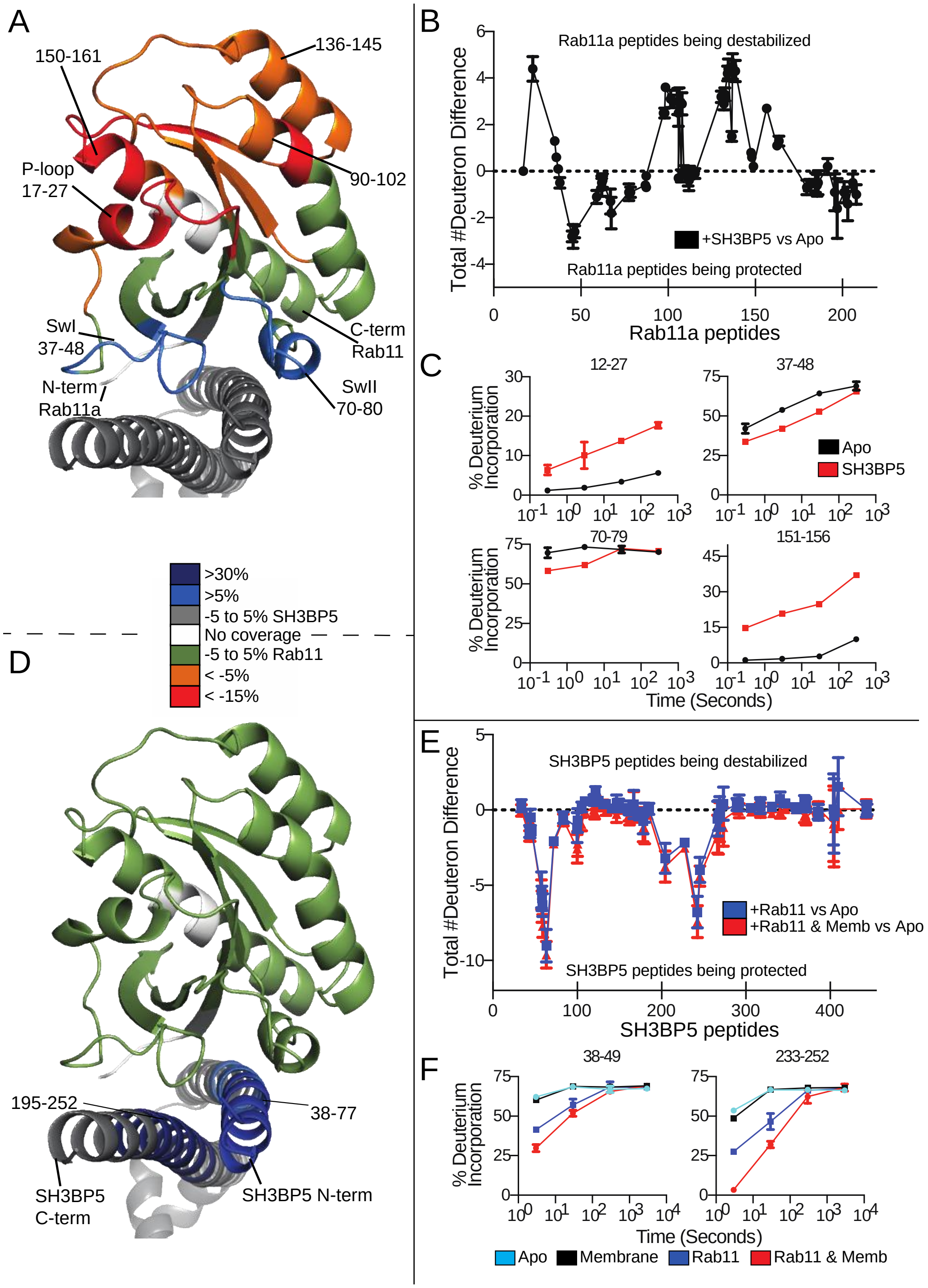

Supplementary Figure 5. HDX-MS Validates the Binding Interface of Rab11 and SH3BP5, and reveals conformational changes in the nucleotide binding pocket.

(A) Peptides with significant changes in deuterium incorporation (both >0.5 Da and >5% at any time point) in the presence of SH3BP5 are mapped on the structure of Rab11 bound to SH3BP5. Differences are mapped according to the legend.

(B) The number of deuteron difference for all peptides analyzed over the entire deuterium exchange time course for Rab11A(Q70L) in the presence of SH3BP5 (31-455). Every point represents the central residue of an individual peptide.

(C) Selected Rab11A peptides that showed decreases and increases in exchange are shown. The full list of all peptides and their deuterium incorporation is shown in Supplementary Data 1.

(D) Peptides with significant changes in deuterium incorporation (both >0.5 Da and >5% at any time point) in the presence of WT Rab11 are mapped on the structure of SH3BP5 bound to Rab11, according to the legend.

(E) The number of deuteron difference for all peptides analyzed over the entire deuterium exchange time course for SH3BP5 (31-455) in the presence of Rab11A (1-211 with a C-terminal His-tag). Experiments were conducted with and without membrane at a final concentration of 0.2mg/ml.

(F) Selected SH3BP5 (31-455) peptides displaying decreases in exchange are shown. The full list of all peptides and their deuterium incorporation is shown in Supplementary Data 1. For all panels, error bars show SD (n=3).

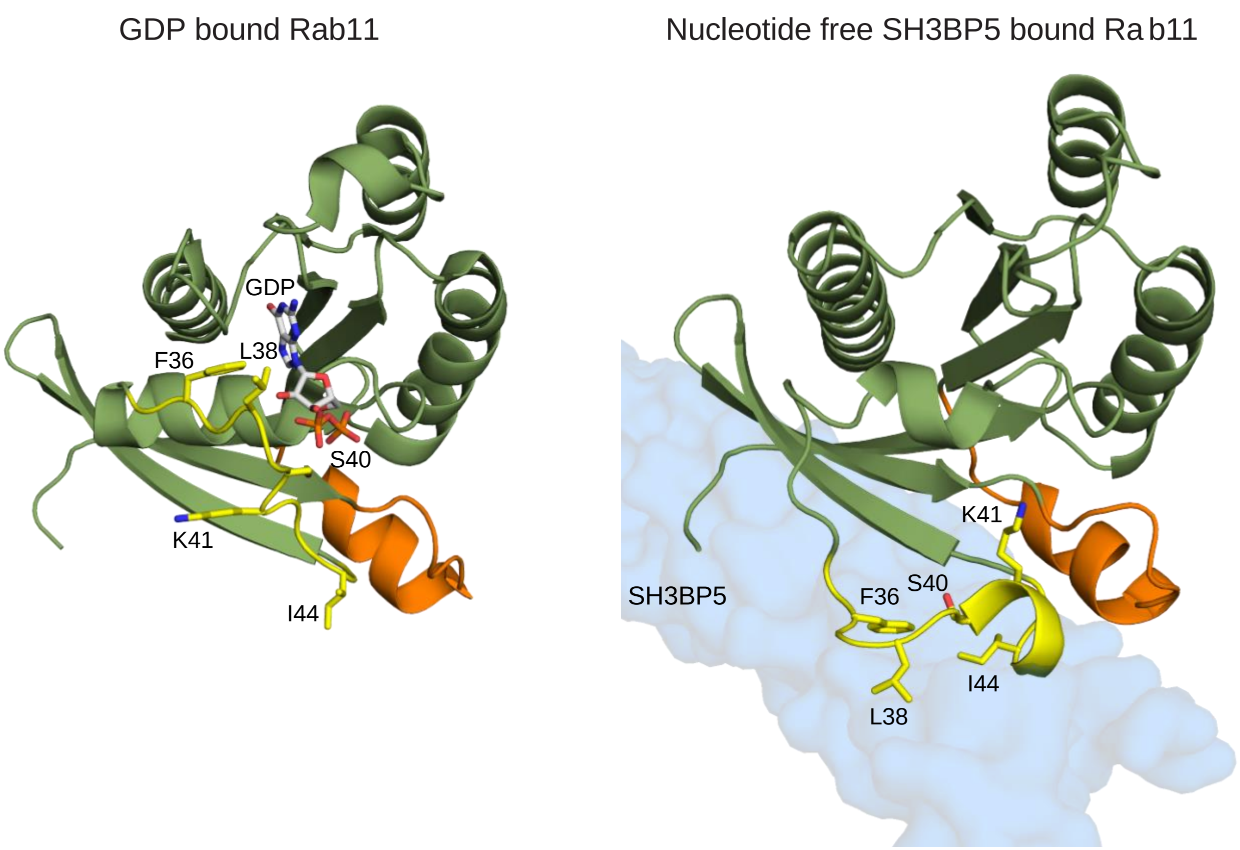

Supplementary Figure 6. Comparison of GDP bound Rab11[3] to nucleotide-free SH3BP5 bound Rab11. Switch I is indicated in yellow, with switch II indicated in orange. Switch I residues that are important in SH3BP5 mediated nucleotide exchange are shown as sticks and labeled on the structure. F36 and L38 interact directly with bound nucleotide, and upon SH3BP5 binding these residues interaction with nucleotide is disrupted, allowing for release of bound nucleotide.

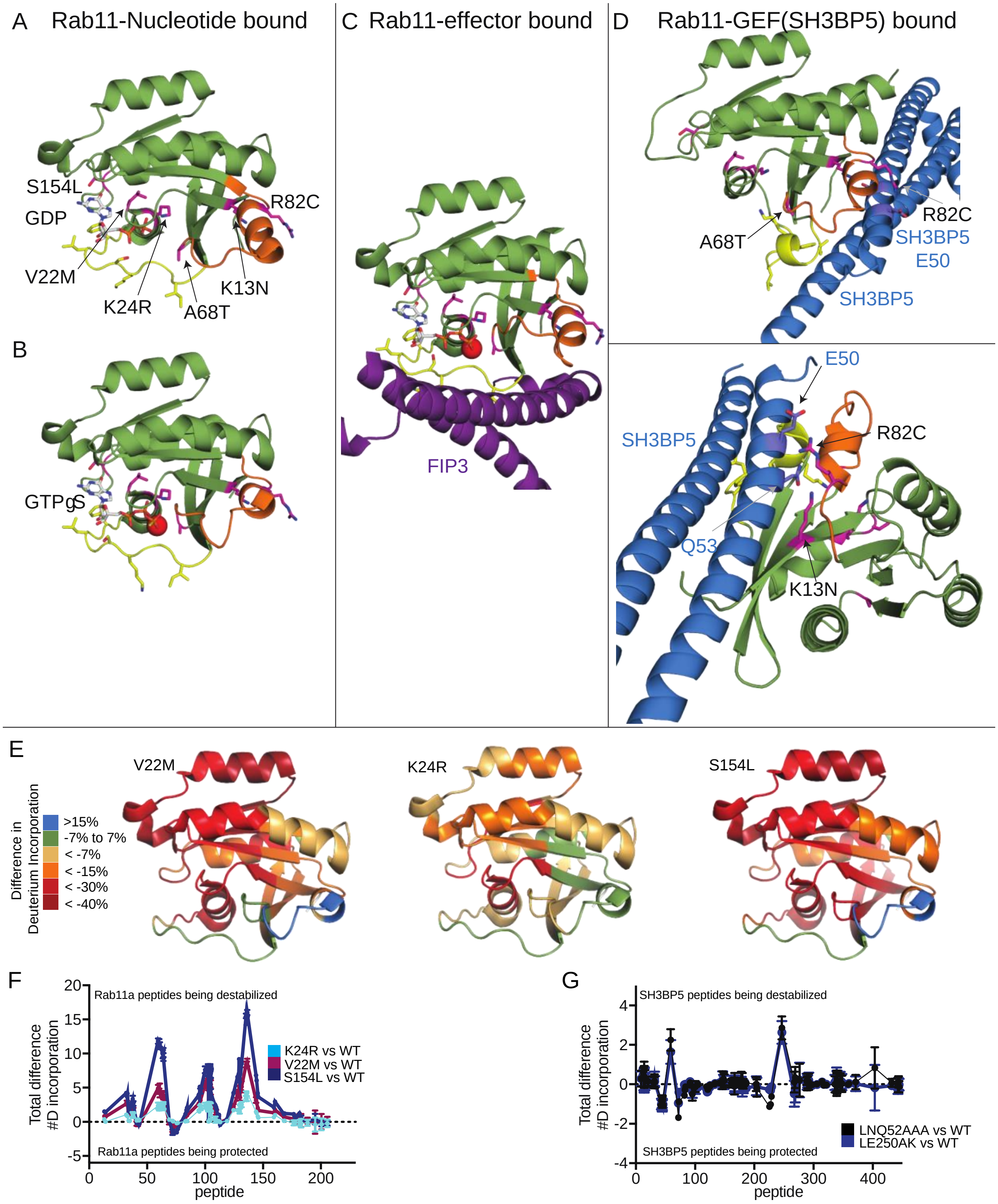
A Rab11-Nucleotide bound
S154L
GDP
R82C
V22M
K13N
K24R
A68T
B
GTPgS
C Rab11-effector bound
FIP3
D Rab11-GEF(SH3BP5) bound
A68T
R82C
SH3BP5
E50
SH3BP5
E50
SH3BP5
R82C
Q53
K13N
E
V22M
K24R
S154L
Difference in Deuterium Incorporation
>15%
-7% to 7%
< -7%
< -15%
< -30%
< -40%
F
Rab11a peptides being destabilized
Rab11a peptides being protected
Total difference #D incorporation
K24R vs WT
V22M vs WT
S154L vs WT
peptide
G
SH3BP5 peptides being destabilized
SH3BP5 peptides being protected
Total difference #D incorporation
LNQ52AAA vs WT
LE250AK vs WT
peptide

158

159

Supplementary Figure 7. Disease linked mutants of Rab11a and Rab11b mapped on the structures of Rab11 bound to nucleotides, effectors, and GEFs.

(A+B) Structure of Rab11 bound to GDP and GTPγS[3] with clinical mutation sites shown in sticks and labeled on the figure.

(C) Structure of Rab11 bound to GTPγS and the Rab11 effector FIP3[4].

(D) Structure of Rab11 bound to SH3BP5, with two views highlighting interaction of Rab11 mutated residues with contact residues in SH3BP5.

(E) HDX-MS experiments comparing WT Rab11 versus V22M, S154L, and K24R. Peptides with significant changes in deuterium incorporation (both >0.5 Da and >5% at any time point) in the mutant are colored on the structure according to the legend. Full HDX experimental data is found in Supplementary Data 1.

(F) The number of deuteron difference for all peptides analyzed over the entire deuterium exchange time course for mutant Rab11 vs WT Rab11.

(G) The number of deuteron difference for all peptides analyzed over the entire deuterium exchange time course for GEF deficient mutations of SH3BP5 (LNQ52AAA and LE250AK) vs WT Rab11. For all panels, error bars show SD (n=3).

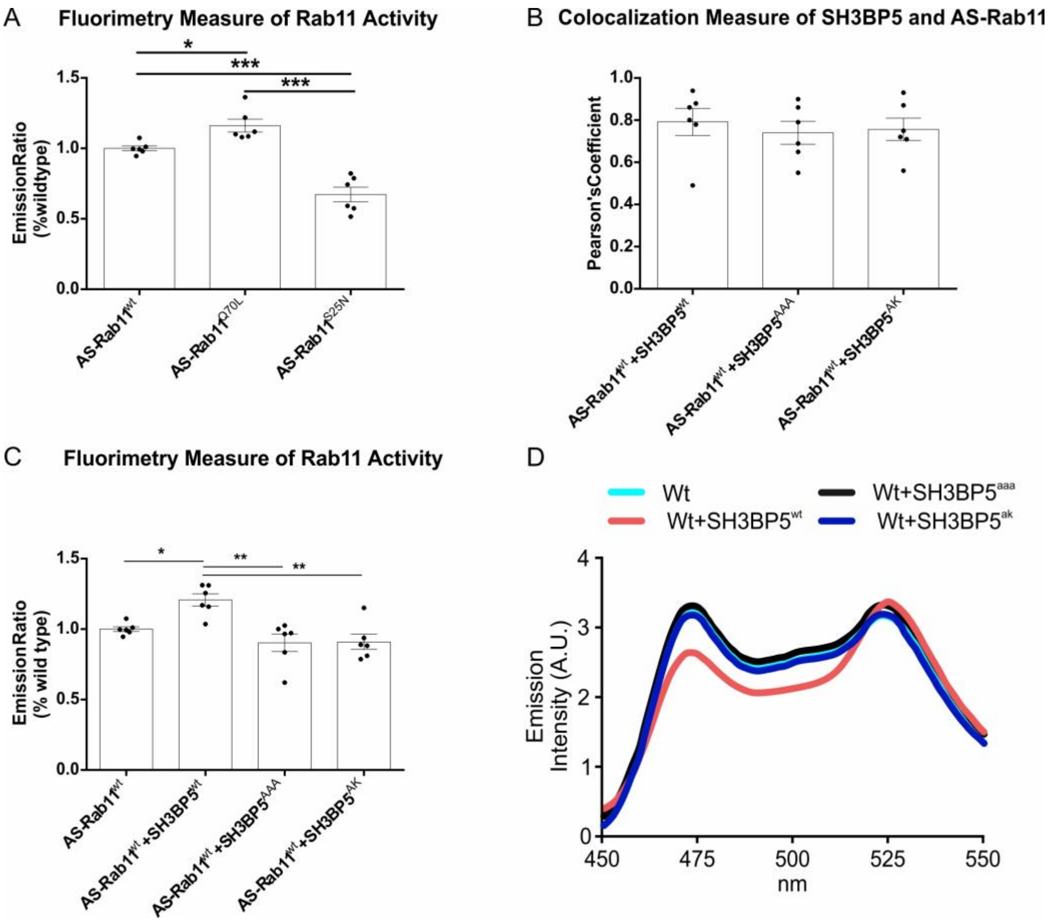

Supplementary Figure 8. AS-Rab11 FRET experiments using dominant active and negative variants of Rab11 and emission spectrum from cellular experiments of Rab11 activation.

(A) Quantification of FRET efficiency of AS-Rab-11^{wt} , constitutively active form (Q70L) or dominant negative (S25N).

(B) Quantification of co-localization of SH3BP5 and total AS-Rab11.

(C). Quantification of FRET efficiency of AS-Rab-11^{wt} upon overexpression of WT SH3BP5, or expression of LNQ52AAA, LE250AK. For all panels, error bars represent SEM (n=6). Significance determined by one-way ANOVA (*=p<0.05, **=p<0.01, ***=p<0.005).

(D) The emission spectrum from excitation at 433 nm for WT AS-Rab11 is shown under either expression of WT-SH3BP5, and GEF deficient SH3BP5 mutants.

Supplementary references

1. Jurrus, E. *et al.* Improvements to the APBS biomolecular solvation software suite. *Protein Sci.* **27,** 112–128 (2018).
2. Afonine, P. V. *et al.* FEM: feature-enhanced map. *Acta Crystallogr. D Biol. Crystallogr.* **71,** 646–666 (2015).
3. Pasqualato, S. *et al.* The structural GDP/GTP cycle of Rab11 reveals a novel interface involved in the dynamics of recycling endosomes. *J. Biol. Chem.* **279,** 11480–11488 (2004).
4. Eathiraj, S., Mishra, A., Prekeris, R. & Lambright, D. G. Structural basis for Rab11-mediated recruitment of FIP3 to recycling endosomes. *Journal of Molecular Biology* **364,** 121–135 (2006).
