# Supplementary material for: Structural determinants of Rab11 activation by the guanine nucleotide exchange factor SH3BP5
Source: Nat Commun. 2018 Sep 14;9:3772. doi: 10.1038/s41467-018-06196-z (PMC6138693; doi:10.1038/s41467-018-06196-z)
Supplement: Supplementary file 3 — Description of Additional Supplementary Files [file 41467_2018_6196_MOESM3_ESM.docx]

Description of Additional Supplementary Files

**File Name:** Supplementary Data 1

**Description:** Summary of all HDX-MS peptide data. Each experiment is displayed on a separate tab of the excel document, with data corresponding to specific figures labeled in the header. The charge state (Z), residue start (S), residue end number (E), and retention time (RT) are displayed for every peptide. Data listed is the average of 3 independent experiments, with SDs presented. Time points are labeled, and the relative level of HDX is colored according to the legend.

**File Name:** Supplementary Movie 1

**Description:** Morph between the GDP bound version of Rab11 and the SH3BP5 bound version of nucleotide-free Rab11 generated using pymol. The movie only represents a simple interpolation between the two states, and does not indicate intermediates in the nucleotide exchange reaction. Switches are colored according to Figure 2, with key residues in switch I shown as sticks. Supplemental Data. Summary of all HDX-MS peptide data. Each experiment is displayed on a separate tab of the excel document, with data corresponding to specific figures labeled in the header. The charge state (Z), residue start (S), residue end number (E), and retention time (RT) are displayed for every peptide. Data listed is the average of 3 independent experiments, with SDs presented. Time points are labeled, and the relative level of HDX is colored according to the legend.
